# Centrin-deficient *Leishmania mexicana* confers protection against Old World visceral leishmaniasis

**DOI:** 10.1038/s41541-022-00574-x

**Published:** 2022-12-03

**Authors:** Subir Karmakar, Greta Volpedo, Wen-Wei Zhang, Patrick Lypaczewski, Nevien Ismail, Fabiano Oliveira, James Oristian, Claudio Meneses, Sreenivas Gannavaram, Shaden Kamhawi, Shinjiro Hamano, Jesus G. Valenzuela, Greg Matlashewski, Abhay R. Satoskar, Ranadhir Dey, Hira L. Nakhasi

**Affiliations:** 1 Divsion of Emerging and Transfusion Transmitted Diseases, CBER, FDA, Silver Spring, MD USA; 2 Department of Pathology and Microbiology, Ohio State University, Columbus, OH USA; 3 Department of Microbiology and Immunology, McGill University, Montreal, QC Canada; 4 Vector Molecular Biology Section, Laboratory of Malaria and Vector Research, National Institute of Allergy and Infectious Diseases, NIH, Rockville, MD 20852 USA; 5 Department of Parasitology, Institute of Tropical Medicine (NEKKEN), Nagasaki University, Nagasaki, Japan; 6 The Joint Usage/Research Center on Tropical Disease, Institute of Tropical Medicine (NEKKEN), Nagasaki University, Nagasaki, Japan

**Keywords:** Live attenuated vaccines, Parasitic infection

## Abstract

Leishmaniasis is one of the top neglected tropical diseases with significant morbidity and mortality in low and middle-income countries (LMIC). However, this disease is also spreading in the developed world. Currently, there is a lack of effective strategies to control this disease. Vaccination can be an effective measure to control leishmaniasis and has the potential to achieve disease elimination. Recently, we have generated *centrin* gene-deleted new world *L. mexicana* (*LmexCen*^*−/−*^) parasites using CRISPR/Cas9 and showed that they protect mice against a homologous *L. mexicana* infection that causes cutaneous disease. In this study, we tested whether *LmexCen*^*−/−*^ parasites can also protect against visceral leishmaniasis caused by *L. donovani* in a hamster model. We showed that immunization with *LmexCen*^*−/−*^ parasites is safe and does not cause lesions. Furthermore, such immunization conferred protection against visceral leishmaniasis caused by a needle-initiated *L. donovani* challenge, as indicated by a significant reduction in the parasite burdens in the spleen and liver as well as reduced mortality. Similar control of parasite burden was also observed against a sand fly mediated *L. donovani* challenge. Importantly, immunization with *LmexCen*^*−/−*^ down-regulated the disease promoting cytokines IL-10 and IL-4 and increased pro-inflammatory cytokine IFN-γ resulting in higher IFN-γ/IL-10 and IFN-γ/IL4 ratios compared to non-immunized animals. *LmexCen*^*−/−*^ immunization also resulted in long-lasting protection against *L. donovani* infection. Taken together, our study demonstrates that immunization with *LmexCen*^*−/−*^ parasites is safe and efficacious against the Old World visceral leishmaniasis.

## Introduction

Leishmaniases are vector-borne parasitic diseases affecting millions of people globally, which clinically range from self-healing cutaneous (CL) to systemic and often fatal visceral leishmaniasis (VL)^1,2^. The various clinical forms of leishmaniases are caused by different parasite species, and CL is caused by the infections with *L. major* or *L. tropica* in the Old World and with *L. mexicana* species complex in the New World. The more severe and life-threatening VL is caused by the *L. donovani* complex in the Old World and by *L. infantum* which is prevalent in the Mediterranean, as well as the New World^3^. Cutaneous leishmaniasis is endemic in the United States, and presence of sand fly vector may cause further spreading of this disease^4,5^.

Since most of the chemotherapies against leishmaniasis suffer from limitations like toxicity, high cost, the necessity for long-term use and most importantly emergence of drug resistance^6–8^, vaccination could be effective in achieving control and elimination of leishmaniasis. Recovery from a primary *Leishmania* infection gives lifelong protection from future infections, thus leading to the insight that vaccination is feasible against leishmaniasis^9–12^. However, there is no licensed vaccine available for human use against any form of leishmaniasis.

Earlier studies have shown that deliberate infections with a low dose of virulent live wild-type dermotropic *L. major* parasites confers protection against reinfection, a process termed leishmanization^13–15^. Leishmanization also afforded cross-protection against VL in various animal models and probably in humans^10,16,17^. However, such method of immunization is not practical because of the safety concerns regarding skin lesions that may last for months at the site of inoculation in a naïve population^18,19^. In contrast, immunization with live attenuated dermotropic *Leishmania* parasites which are non-pathogenic and provide a complete array of antigens like their wild-type analogs, could be a promising vaccine candidate against both CL and VL. While leishmanization with the Old World species of *L. major* is widely studied to identify the mediators of protective immunity and used as a standard to replicate in numerous experimental vaccine studies, there is no equivalent leishmanization with the New World species of *Leishmania*, such as *L. mexicana*. Infections with *L. mexicana* species cause a more chronic pathology that, unlike skin lesions caused by *L. major* infection, does not self-resolve. Further, infection with *L. mexicana* presents clinical features and pathologies distinct from the infections of Old World *Leishmania* species. For example, the localized cutaneous lesions caused by *L. mexicana* infection, can progress into diffuse lesions in other parts of the body away with no delayed-type hypersensitivity (DTH) response^20,21^. In addition, induction of a Th2 dominant response is responsible for pathogenesis in *L. mexicana* infection of mice^22^. A similar dominant Th2 response has been shown to play an important role in the pathogenesis of a related New World species, *L. braziliensis*, in a hamster model^23^. Therefore, it is necessary to develop vaccines that are effective at preventing infections with the New World *Leishmania* species. We recently demonstrated the safety and efficacy of a marker-free *centrin* gene-deleted live attenuated new world dermotropic *L. mexicana* (*LmexCen*^*−/−*^) parasite in a mouse model of New World cutaneous leishmaniasis^24^. Centrin is a calcium-binding protein, essential in the duplication of centrosomes in eukaryotes including *Leishmania*^25,26^. *Centrin* gene-deficient *Leishmania* parasites are replication-deficient only in the intracellular amastigote stage but can be easily grown in promastigote culture^25^. Similar deletion of centrin in *L. major* (*LmCen*^*−/−*^) showed that immunization with this strain derived from the Old World species of *Leishmania* can protect against CL and VL challenge infections^27,28^.

The current study examined whether a New World dermotropic *LmexCen*^*−/−*^ parasite is similarly effective against fatal VL in a hamster model. Hamsters develop clinicopathological symptoms of VL similar to human VL, including succumbing to death and are considered a gold standard model of VL^29^. Data showed that immunization of hamsters with live attenuated *LmexCen*^*−/−*^ is safe and cross-protects against a *Leishmania donovani* challenge infection. Moreover, *LmexCen*^−/−^ vaccinated hamsters showed downregulation of disease promoting Th2 type of response as indicated by reduced IL-10 and IL-4 cytokine expression and higher IFN-γ response resulting in higher IFN-γ/IL-10 and IFN-γ/IL-4 ratios, the key biomarkers of protection compared to virulent *LmexWT* infection. Further, immunization with *LmexCen*^*−/−*^ resulted in long-lasting protection against *L. donovani* infection through needle injection. These studies show that immunization with genetically modified New World *Leishmania mexicana* parasites has the potential as a vaccine against the New World *Leishmania* species *L. mexicana*^24^ and also against the Old World visceral leishmaniasis caused by *L. don*ovani.

## Results

### *LmexCen*^*−/−*^ parasites do not cause any lesion development in hamsters

**Fig. 1:**
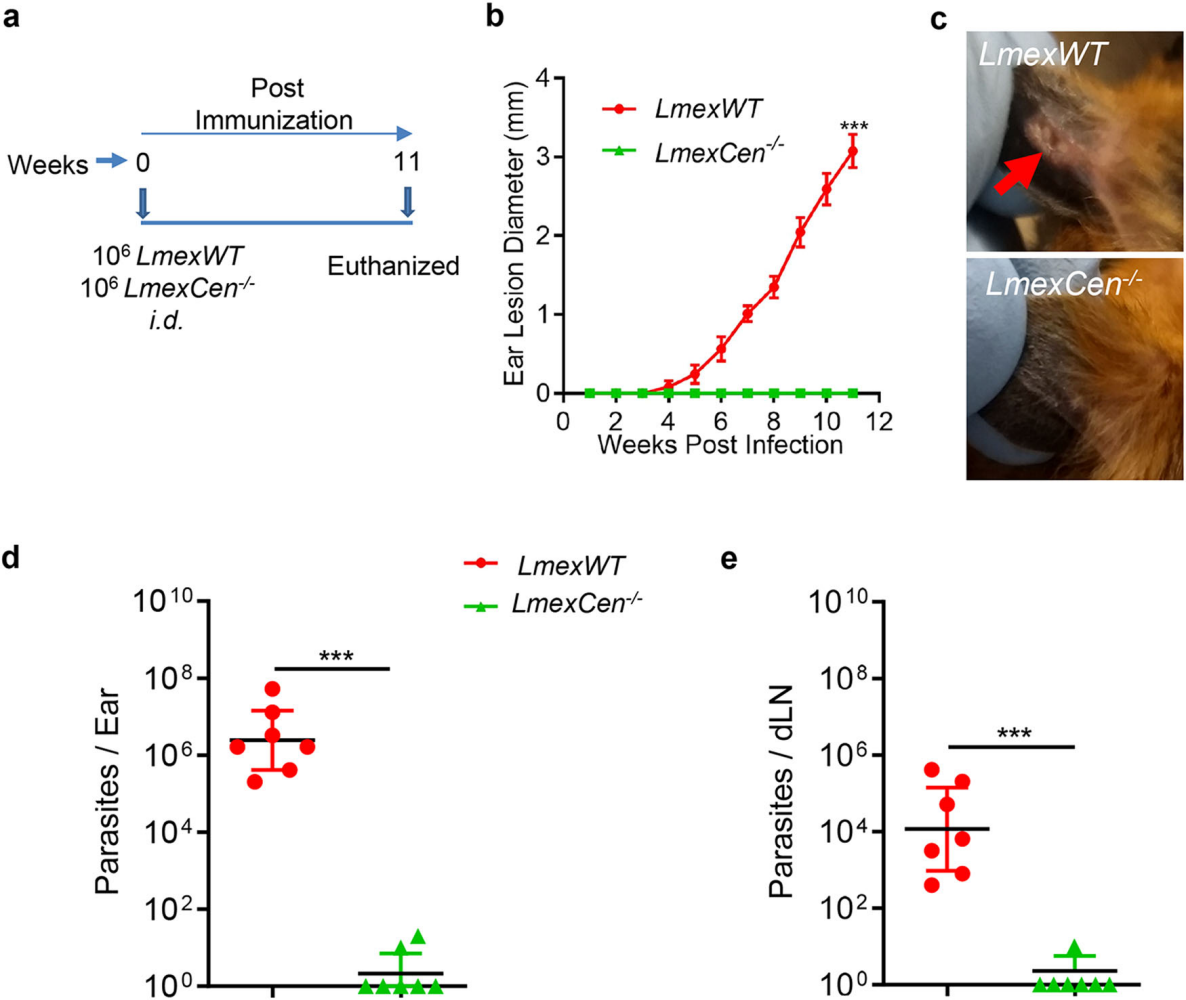
Live attenuated *LmexCen*^*−/−*^ parasites are safe and do not cause lesions in hamster. **a** Schematic representation of the experimental plan. **b** Lesion size was monitored every week in hamsters injected with 10^6^-total stationary phase either *LmexWT* or *LmexCen*^*−/−*^ parasites by intradermal (i.d) injection. Ear lesion diameters were measured at indicated weeks post inoculation. Results (SEM) are representative cumulative effect of two independent experiments, 1 ear, total 7 hamsters per group (***p = 0.0006 values were determined by Mann–Whitney two-tailed test). **c** Photographs of representative ears of *LmexWT* and *LmexCen*^*−/−*^ immunized hamsters at 11 weeks post inoculation (PI). Red arrow indicates the lesion development. Parasites load in the ear (**d**) and draining lymph node (dLN) (**e**) of *LmexWT* and *LmexCen*^*−/−*^ immunized hamsters were determined by serial dilution assay at 11 weeks post inoculation. Results (the geometric means with 95% Cl) represent cumulative of two independent experiments; in first experiment *n* = 4/group and in second experiment *n* = 3/group (***p = 0.0006 values were determined by Mann–Whitney two-tailed test).

To evaluate the non-pathogenicity of *LmexCen*^*−/−*^ parasites, hamsters were injected intradermally with 10^6^ *LmexCen*^*−/−*^ promastigotes. Hamsters were injected with wild-type *L. mexicana* (10^6^, *LmexWT*) parasites were used as a control group. Lesion development was monitored up to 11-weeks post-injection, and parasite loads were determined at this study endpoint through the serial dilution method (Fig. 1a). The hamsters were injected with *LmexCen*^*−/−*^ parasites did not develop any visible lesions up to 11-weeks of post-injection (Fig. 1b, c). In contrast, the hamsters injected with *LmexWT* parasites developed ear lesions within 5-weeks of injection that progressively increased in size (Fig. 1b, c). The *LmexWT*-injected hamsters had significantly higher parasite loads both in the ears (~10^6^ Fig. 1d) and in the dLNs (~10^4^ Fig. 1e) compared to low levels of viable parasites were recovered from the ears (2 out of 7) and draining lymph nodes (1 of 7) of (Fig. 1d, e) *LmexCen*^*−/−*^ immunized hamsters. No viable parasites were recovered from the spleen, liver, or bone marrow of either *LmexCen*^*−/−*^ or *LmexWT* injected hamsters at 11-weeks post-inoculation). These results showed that *LmexCen*^*−/−*^ parasites are non-pathogenic, thus safe.

### Immunization with *LmexCen*^*−/−*^ induces a pro-inflammatory immune response

**Fig. 2:**
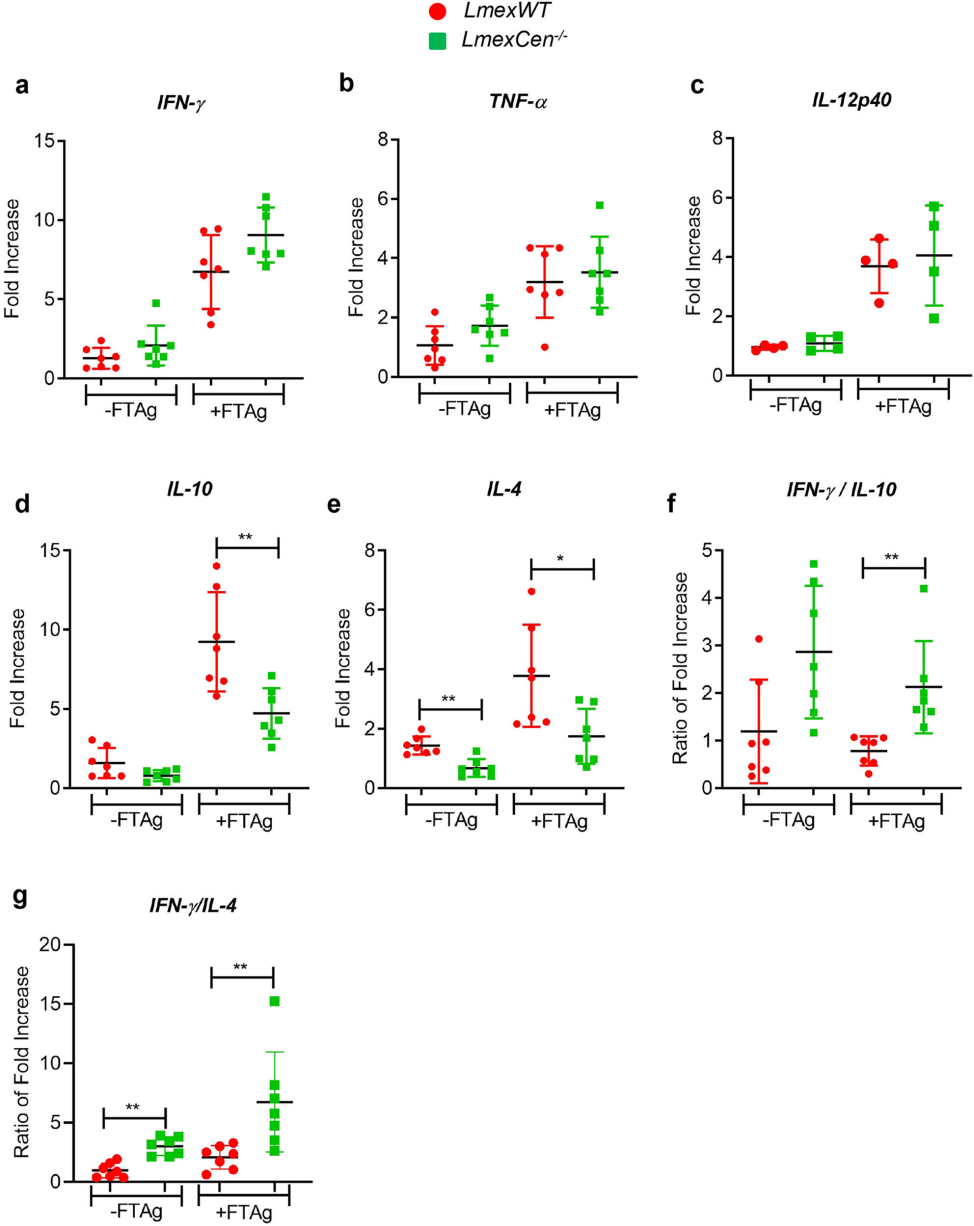
Live attenuated *LmexCen*^*−/−*^ parasites down regulate disease promoting cytokines expression in spleen of hamsters. **a** IFN-γ, **b** TNF-α, **c** IL-12p40, **d** IL-10, and **e** IL-4 expression in the spleen of *LmexWT* or *LmexCen*^*−/−*^ inoculated hamsters was evaluated by RT-PCR following 11 weeks post-inoculation. The ratio of IFN-γ/IL-10 (**f**) and IFN-γ/IL-4 (**g**) expression in the spleen was also determined. Except IL-12p40, results (mean ± SD) are cumulative of two independent experiments; in first experiment *n* = 4/group and in second experiment *n* = 3/group. Statistical analysis was performed by Mann–Whitney two-tailed test (*p = 0.0175, and **p = 0.007).

Next, we evaluated the immune response associated with the immunization by analyzing the expression of major proinflammatory (IFN-γ) and disease promoting (IL-10 and IL-4) cytokines in the splenocytes of *LmexCen*^*−/−*^ and *LmexWT* injected hamsters at 11 weeks post-infection (Fig. 2). To measure antigen specific immune response, splenocytes were either unstimulated or stimulated with *L. mexicana* freeze thawed antigen (±FTAg). In both *LmexCen*^*−/−*^ immunized and *LmexWT* infected animals, upon antigen stimulation, expression of IFN-γ increased in the spleen even though the increase was not statistically significant between the two groups (Fig. 2a). Of note, the parasite burden was significantly different between the two groups (Fig. 1 from parasite burden). We also measured other inflammatory cytokines TNF-α and IL-12p40 at this time point (Fig. 2b, c). Our data indicate no significant difference in their expression between *LmexWT* and *LmexCen*^*−/−*^ immunized groups. Measurement of anti- disease promoting cytokines IL-10 and IL-4, on the other hand, revealed contrasting results. In the spleens of *LmexCen*^*−/−*^ immunized hamsters both the IL-10 and IL-4 expression was significantly inhibited compared to *LmexWT* infected group (Fig. 2d, e). Moreover, the ratios of IFN-γ to IL-10 as well as IFN-γ to IL-4 were significantly higher in the spleens of the *LmexCen*^*−/−*^ immunized group compared to *LmexWT* injected group(Fig. 2f, g), suggesting that *LmexCen*^*−/−*^ immunization induces a pro-inflammatory environment while suppressing anti-inflammatory response in the spleens of immunized animals. Collectively, these results demonstrated that the live attenuated *LmexCen*^*−/−*^ parasites are immunogenic in a hamster model without causing cutaneous lesions.

### Immunization with *LmexCen*^*−/−*^ protects against challenge infection with *L. donovani* through needle and sand fly mediated infection in a hamster model

**Fig. 3:**
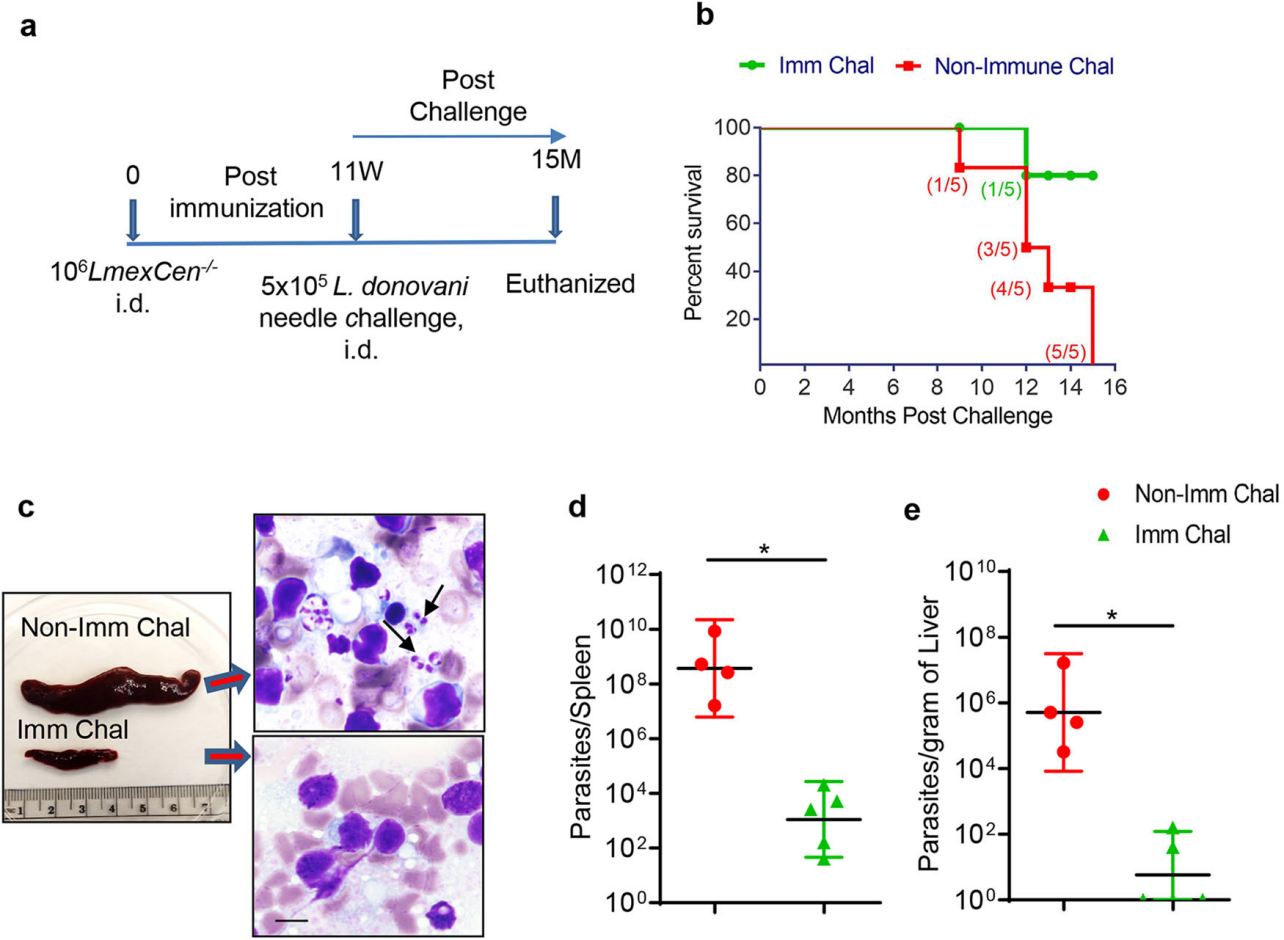
*LmexCen*^*−/−*^ immunization confers protection against *L. donovani* through needle challenge in hamsters. **a** Schematic representation of the experimental plan to determine the protection efficacy of *LmexCen*^*−/−*^ parasite. **b** Kaplan–Meier survival curves of *LmexCen*^*−/−*^ immunized hamsters (Imm Chal; green lines, *n* = 5) following challenge with *L. donovani* and compared with age-matched non-immunized challenged group (Non Imm Chal; red lines, *n* = 5). **c** Photographs (left panel) of representative one spleen samples of both *LmexCen*^*−/−*^*-*immunized and non-immunized hamsters following 12-months of post-needle challenge hamster is shown. Right panel showing stamp smear of respective spleens stained with H&E, black arrows indicates intracellular parasites (bar-20 µm). Parasite loads in the spleen (**d**) and liver (**e**) of hamsters either immunized with *LmexCen*^*−/−*^ (Imm Chal, *n* = 5) parasites or age-matched non-immunized control (Non-Imm Chal, *n* = 4) were determined by serial dilution method following 9–15 months of post-needle challenge with *L. donovani*. Results (the geometric means with 95% Cl) are from one experiment. Statistical analysis (*p = 0.01) was performed by Mann–Whitney two-tailed test.

Since *LmexCen*^*−/−*^ parasites are immunogenic in hamsters, we investigated the efficacy of *LmexCen*^*−/−*^ immunization against challenge infection with *L. donovani*. The hamsters were intradermally immunized with *LmexCen*^*−/−*^ parasites. Immunized hamsters were challenged at 11-weeks post-immunization with virulent *L. donovani* wild-type (*LdWT*) parasites and monitored up to 15-months post-challenge (Fig. 3a). Survival data revealed that *LmexCen*^*−/−*^ immunization protected 80% of animals against mortality associated with *L. donovani* infection, as demonstrated by the survival of 4 out of the 5 animals that remained healthy up to 15-months post-challenge, the endpoint of our study. All the age-matched non-immunized- hamsters died with symptoms characteristic of human VL starting from 9-months post-challenge timepoint and up to 15-months corresponding to the endpoint of the study (Fig. 3b). Spleens isolated from the moribund hamsters from the non-immunized challenged group demonstrated splenomegaly (Fig. 3c). In contrast, spleens from *LmexCen*^*−/−*^ immunized challenged group showed no splenomegaly consistent with a lack of pathology in this group. Moreover, analysis of the parasite burden revealed significant control of parasite burden both in the spleen (~5 log reduction, Fig. 3d) and liver tissues (~4 log reduction, Fig. 3e) of *LmexCen*^*−/−*^ immunized hamsters compared to the nonimmunized-challenged group. Of note, 60% (3 out of 5) of the livers from immunized animals had no detectable parasites as measured by serial dilution method.

**Fig. 4:**
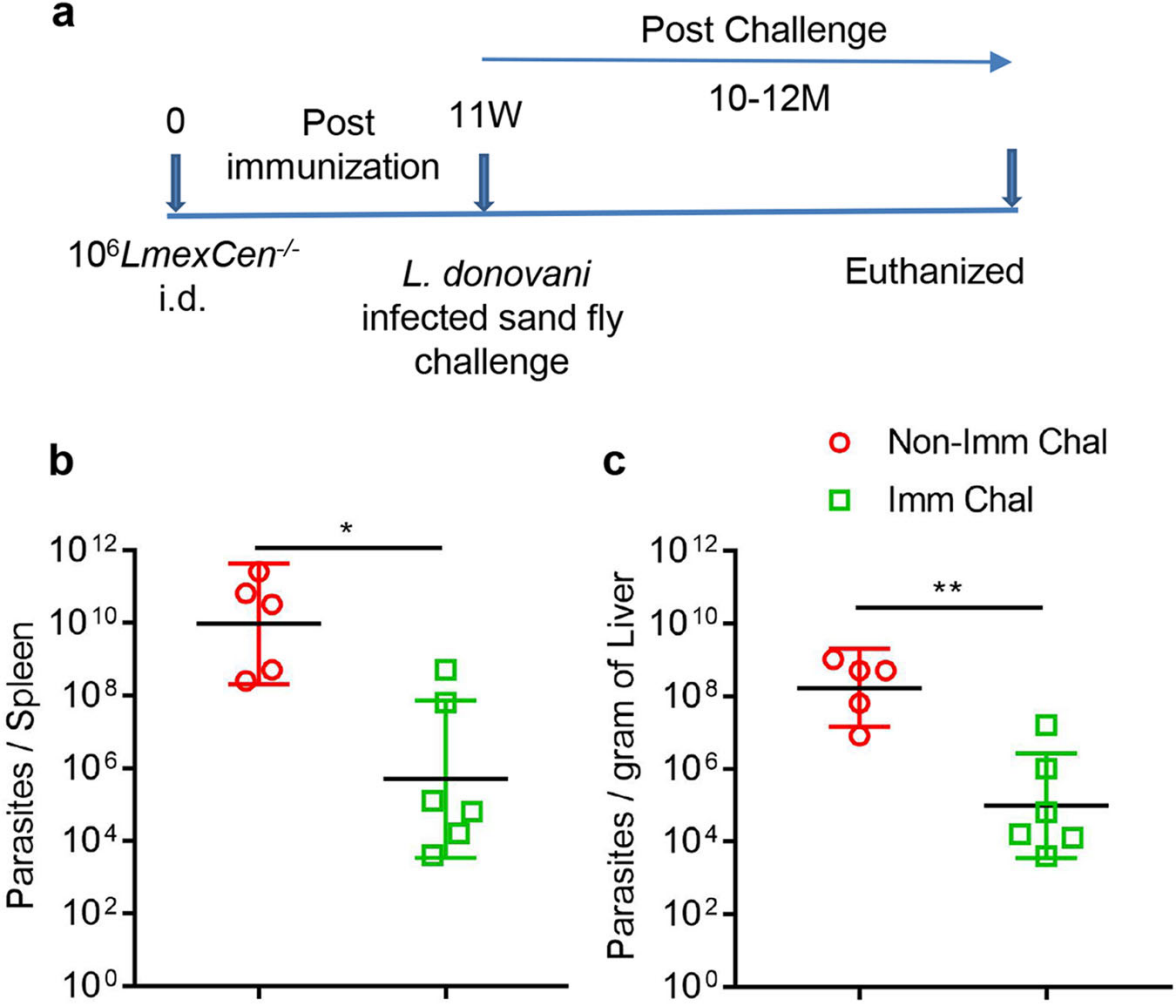
*LmexCen*^*−/−*^ immunization confers protection against sand fly transmitted *L. donovani* infection in hamsters. **a** Schematic representation of the experimental plan. Parasite load in the spleen (**b**) and liver (**c**) of age matched non-immunized (Non-Imm chal, *n* = 5) and *LmexCen*^*−/−*^ *-*immunized (Imm chal, *n* = 6) hamsters were determined after 10–12 months post *L. donovani* infected sand fly challenge. Results represent (the geometric means with 95% Cl) one experiment (*p = 0.01 and **p = 0.008 values were determined by Mann–Whitney two-tailed test). Results are from one experiment.

To confirm if immunization with *LmexCen*^*−/−*^ can similarly protect the hamsters against a more rigorous challenge with a sand fly mediated *L. donovani* infection, immunized hamsters were exposed to *L. donovani* infected-sand flies at 11-weeks post-immunization and monitored for 12-months post-challenge (Fig. 4a). The animals were sacrificed at 10 to12-months, and parasite burden in spleens and livers was determined. There was a significant reduction of parasite burden in both spleen and liver of the immunized hamsters compared to non-immunized animals (~4 log reduction Fig. 4b, c). Together, these data demonstrate that *LmexCen*^*−/−*^ immunization protects against both needle and sand fly mediated infection in a hamster model of VL, suggesting that immunization with the New World CL vaccine can also protect against the Old World VL.

### Immunization with *LmexCen*^*−/−*^ confers long-term protection against fatal visceral infection in a hamster model

**Fig. 5:**
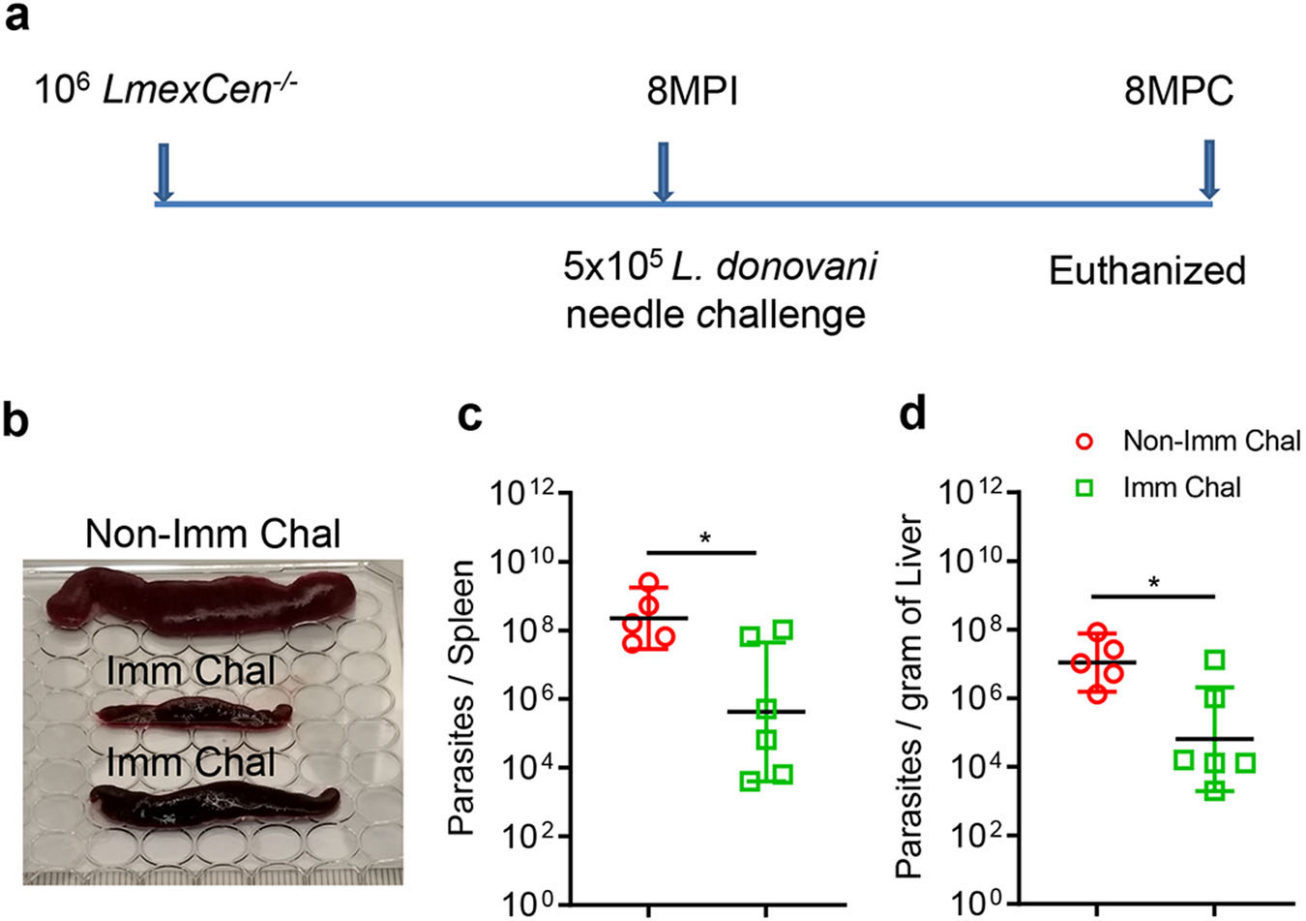
*LmexCen*^*−/−*^ immunization confers long term protection against needle challenge *L. donovani* infection in hamsters. **a** Schematic representation of experimental plan to determine the long-term protective efficacy of *LmexCen*^*−/−*^ parasites against *L. donovani* infection through needle injection. **b** Representative spleen picture from each group. Spleen (**c**) and Liver (**d**) parasite burden of *LmexCen*^*−/−*^ immunized (Imm Chal, *n* = 6) and age matched non-immunized (Non-Imm Chal, *n* = 5) hamsters were determined at 8 months post needle challenge. Results (the geometric means with 95% Cl) are from one experiment (*p = 0.03 values were determined by Mann–Whitney two-tailed test). MPI Months post-immunization, MPC months post-challenge.

Next, to determine the durability of protection induced by *LmexCen*^*−/−*^ immunization, hamsters were challenged with the virulent wild-type *L. donovani* through a needle injection 8-months post-immunization (Fig. 5a). Since we observed comparable protection to both the needle and the sand fly mediated challenges (Figs. 3 and 4), in this experiment only needle challenged was used. Age-matched naïve *L. donovani* infected animals were used as a control group (non-immune challenged group). Analysis of spleen and liver parasite loads after 8-months post-challenge showed significant control of spleen and liver parasite burden. The immunized hamsters showed lack of splenomegaly (Fig. 5b) and a ∼2 and a ∼1.5 log-fold reduction in the spleen (Fig. 5c) and liver (Fig. 5d) parasite burden, respectively, compared to non-immunized challenged hamsters. These data confirm the long-lasting protective immunity of *LmexCen*^*−/−*^ parasites against visceral infection in the preclinical hamster model.

## Discussion

Antileishmanial treatment is complex and shows toxic side effects. Therefore, a vaccine is urgently needed against human leishmaniasis, VL in particular. Epidemiological evidence and laboratory studies suggested that infection with one species of *Leishmania* species can cross-protect against other species^10,16,17,30^. Particularly, recovery from infection with the Old World species such as *L. major* parasites has been shown to confer protection against New World species *L. infantum*, which causes fatal visceral leishmaniasis, confirming that leishmanization is a viable vaccination approach^16^. Although, infection with *Leishmania mexicana* induces skin pathology similar to *L. major* infection, may also result in the development of non-healing lesions in contrast to the spontaneously healing lesions observed in *L. major* infections^20,21^. In addition, *L. mexicana* infection is associated with a dominant Th2 response as compared to *L. major* parasites^22^, whereas the role of Th2 response in the pathogenesis caused by *L. major* is debatable^31,32^. Therefore, due to the differences in clinical severity and in the immunological mechanisms of pathogenesis, it may be important to develop a vaccine that can protect against infections of *Leishmania* species prevalent in the New World. Towards that goal, we have developed *centrin* gene-deleted *L. mexicana* parasites using the CRISPR/Cas9 technique. Our studies in mouse infection models indicated that *LmexCen*^*−/−*^ immunization confers protection against homologous wild-type *L. mexicana* challenge infection^24^. Since both the CL and the VL are prevalent in the Americas, a vaccine against these diseases will be needed. Therefore, we wanted to test the hypothesis whether a New World strain derived *LmexCen*^*−/−*^ can also protect against a VL infection.

This study reports that *LmexCen*^*−/−*^ parasites induce significant host immune response and protect hamsters from developing severe VL disease upon challenge with the Old World *L. donovani* parasites. Our results show that *LmexCen*^*−/−*^ parasites do not cause any pathology in susceptible hosts such as hamsters^33^. Our data further confirmed the safety characteristics of *LmexCen*^*−/−*^ parasites in the hamster model, as was previously shown in mouse models of infection^24^. Immunization with *LmexCen*^*−/−*^ showed downregulation of the disease promoting cytokine response in hamster spleens compared to the spleens of *LmexWT*-infected animals, as was also observed in mouse models of infection^24^. Specifically, while *LmexWT* inoculated hamsters developed a disease promoting cytokine response, as evident by the elevated IL-10 and IL-4 expression levels, *LmexCen*^***−/−***^ immunized hamsters displayed a diminished expression of both these cytokines relative to *LmexWT* infection. The hamster is considered as a gold standard model to study visceral leishmaniasis because it elicits all the clinical features of human disease as well as the expression profile of different cytokines related to disease progression or control were identified. Like humans, in hamsters IL-10 helps in disease progression and corelates with VL, while expression of IL-4 is unaltered^34^. However, it is well documented that IL-4 levels were high in active VL patients^35,36^. On the other hand, there was similar induction of pro-inflammatory cytokine IFN-γ, ΤΝF−α and IL−12 between *LmexWT* infected and *LmexCen*^***−/−***^ immunized hamster spleens despite the significant difference in parasite burden in these groups. Overall, analysis of splenic immune response showed the ratios between IFN-γ/IL-10 and IFN-γ/ IL-4 higher in *LmexCen*^*−/−*^ immunized hamsters than in *LmexWT* infection. In mice infected with *L. major*, IL-4 can play a critical role in modulating the establishment of a protective Th1 response^31,37^. However, in *L. mexicana* infection, both IL-10 and IL-4 play a crucial role in disease susceptibility in mouse models^38,39^. In contrast, *LmCen*^*−/−*^ immunization induced a dominant Th1 response (60 folds high IFN-γ compared to *LmWT*) evident throughout the immunization period^27^. However, IL-10 and IL-4 responses were significantly diminished in *LmexCen*^*−/−*^ immunization (6 folds enrichment of IFN-γ/IL-10 in *LmCen*^*−/−*^ versus 2-3 folds enrichment in *LmexCen*^*−/−*^), indicating that *LmCen*^*−/−*^ induces an exaggerated immune response when tested at an equivalent timepoint post-immunization in hamsters. It is also well established that in *L. mexicana* infection, a diminished expression of Th2 response is crucial to provide host protection^40,41^. Our current studies suggest that immunization with the *LmexCen*^***−/−***^ parasites in both rodent models induced a similar protective immune response^24^.

Next, we observed that the immune response generated by *LmexCen*^***−/−***^ immunization induced significant host protection against *L. donovani* challenge both in the spleen and liver up to 15- months of post-challenge, as evident by the substantial reduction in parasite burden compared to non-immunized animals. In contrast, all non-immunized challenged hamsters developed severe symptoms of VL and succumbed to death by 15-months of post-challenge. On the contrary, 80% of immunized and challenged animals survived until the study ended. In addition, *LmexCen*^***−/−***^ induced immunity was long-lasting, as evidenced by the parasite control in animals immunized over eight months compared to non-immunized animals. This could be due to the persistence of vaccine parasites in immunized animals that may play an essential role in maintaining a protective immune response. In support of that, we observed a small number of live vaccine parasites recovered from some of the hamsters even after 11-weeks post-immunization (Fig. 1) that could maintain immunity as was observed in our previous studies with the *LmCen−/−* parasites^27^.

In conclusion, we have demonstrated that using CRISPR/Cas9 mediated *centrin* gene deletion of *L. mexicana* parasite, a New World *Leishmania* species, is safe and effective against VL caused by the Old World species, *L. donovani*. Furthermore, these studies demonstrate that centrin deleted parasites, whether from the Old World or the New World, *Leishmania* offer an expanded number of vaccine candidates that could be developed to control leishmaniasis in all endemic regions of the world and eventually eliminate the disease. However, there are challenges with the *Leishmania* vaccines with respect to moving it from bench to bed side in resource poor countries that are *Leishmania* endemic as was pointed out by Parkash et al.^42^. We are currently working on some of such challenges.

## Methods

### Study design and ethical statement

Immunization and challenge infections were performed in a hamster model of VL to determine the efficacy of *centrin* gene-deleted *L. mexicana* (*LmexCen*^*−/−*^) parasites as a vaccine against experimental *L. donovani* infection. All animal experiments in this study were reviewed and approved by the Animal Care and Use Committee of the Center for Biologics Evaluation and Research, U.S. Food and Drug Administration (ASP 1999#23) and the National Institute of Allergy and Infectious Diseases (NIAID) (http://grants.nih.gov/grants/olaw/references/phspolicylabanimals.pdf) under animal protocol LMVR4E. The NIAID DIR Animal Care and Use Program complies with the Guide for the Care and Use of Laboratory Animals and with the NIH Office of Animal Care and Use and Animal Research Advisory Committee guidelines. The housing condition of animals were followed standard guidelines by NIH guidelines for the humane care and use of animals. The body weight, fur and physical appearance was monitored and periodically assessed by the facility veterinarian to assess the development of VL. Sample sizes were calculated as the following: 4 hamsters per group can provide over 80% power to detect a 2-fold difference on parasite load (survival) averaged across the tested time points comparing the hamsters with and without the immunization (α = 0.05 and CV = 30%).

### Animals and parasites

Six to eight-week-old female outbred Syrian golden hamsters (*Mesocricetus auratus*) were obtained from the Harlan Laboratories Indianapolis, USA. All animals were housed either at the Food and Drug Administration (FDA) animal facility, Silver Spring (MD) or the National Institute of Allergy and Infectious Diseases (NIAID), Twin-brook campus animal facility, Rockville (MD), under pathogen-free conditions. The wild-type *L. donovani* (*LdWT*) (MHOM/SD/62/1S) parasites, wild-type *Leishmania mexicana* (MNYC/B2/62/m379) parasites (*LmexWT*) and *centrin* gene-deleted *LmexCen*^*−/−*^ promastigotes were cultured in liquid M199 culture medium supplemented with 10% fetal bovine serum (FBS), 1% Penicillin/Streptomycin and 1% HEPES at 26 C°^24,27^.

### Immunization of hamsters and determination of parasite load

Six to eight-week-old female hamsters were immunized with 10^6^ total stationary-phase *LmexCen*^*−/−*^ parasites by intradermal injection in the left ear in 10 μl PBS using a 29-gauge needle (BD Ultra-Fine). In addition, the age-matched control group of animals were infected with 10^6^ total stationary-phase *L. mexicana* wild-type (*LmexWT*) promastigotes. Lesion size was monitored weekly by measuring the diameter of the ear lesion using a direct reading vernier calliper. Parasite burdens in the ear, draining lymph node (dLN), spleen, liver and bone marrow tissues were determined by limiting dilution assay^27^. Ear tissues were treated with Liberase enzyme (Sigma-Aldrich) (0.15 mg/ml) in DMEM mediun at 37 °C. After 90 min of incubation tissues were grinded to make single cell suspensison using BD medimachine system. Cells were washed two times with M199 medium. Lymph nodes, spleen, liver and bone marrow were harvested and homogenized with a cell strainer in 2 ml of M199 medium supplemented with 10% FBS and 1% Penicillin/Streptomycin. To determine parasite burden cell suspension from each organs were serially diluted in 96 well tissue culture plates. After 12 days of incubation at 26 °C, plates were examined with a inverted microscope and parasite burden were calculated for each organ.

### Determination of cytokine expression in splenocytes by real-time PCR

Single cell suspensions from the spleens of hamsters were made at eleven weeks post-immunization with *LmexCen*^*−/−*^ as well as infected with *LmexWT*. The spleen cell suspensions were stimulated with *L. mexicana* freeze-thawed antigen (FTAg), and total RNA was extracted using PureLink RNA Mini kit (Ambion) at 16 h after restimulation. Aliquots (400 ng) of total RNA were reverse transcribed into cDNA by a high-capacity cDNA reverse transcription kit using random hexamers (Applied Biosystems). Cytokine gene expression levels were determined by TaqMan PCR probe (TaqMan, Universal PCR Master Mix, Applied Biosystems) using a CFX96 Touch Real-Time System (BioRad, Hercules, CA). The sequences of the primers (forward and reverse) and probes (5′ 6-FAM and 3′ TAMRA Quencher) were used to detect the gene expression, Table 1^27^. The data were analyzed with CFX Manager Software. The expression levels of genes of interest were determined by the 2^−ΔΔCt^ method; samples were normalized to γ-actin expression and determined relative to expression values from naive hamsters.

**Table 1:** Primers and probes used to detect expression of the cytokines.

Hamster target gene	Primers and probes sequences	
IL-12p40	Forward	5’-AATGCGAGGCAGCAAATTACTC-3'
	Reverse	5’-CTGCTCTTGACGTTGAACTTCAAG-3'
	Probe	5’-(6FAM)-CCTGCTGGTGGCTGACTGCAATCA-(TAMRA)-3'
IFN-γ	Forward	5’-TGTTGCTCTGCCTCACTCAGG-3'
	Reverse	5’-AAGACGAGGTCCCCTCCATTC-3'
	Probe	5’-(6FAM) TGGCTGCTACTGCCAGGGCACACTC-(TAMRA)-3'
TNF-α	Forward	5’-TGAGCCATCGTGCCAATG-3'
	Reverse	5’-AGCCCGTCTGCTGGTATCAC-3'
	Probe	5’-(6FAM)-CGGCATGTCTCTCAAAGACAACCAG-(TAMRA)-3'
IL-4	Forward	5’-ACAGAAAAAGGGACACCATGCA-3'
	Reverse	5’-GAAGCCCTGCAGATGAGGTCT-3'
	Probe	5’-(6FAM) AGACGCCCTTTCAGCAAGGAAGAACTCC-(TAMRA)-3'
IL-10	Forward	5’-GGTTGCCAAACCTTATCAGAAATG-3'
	Reverse	5’-TTCACCTGTTCCACAGCCTTG-3'
	Probe	5’-(6FAM) TGCAGCGCTGTCATCGATTTCTCCC-(TAMRA)-3'
γ-actin	Forward	5’-ACAGAGAGAAGATGACGCAGATAATG-3'
	Reverse	5’-GCCTGAATGGCCACGTACA-3'
	Probe	5’-VIC -TTGAAACCTTCAACACCCCAGCC-(TAMRA)-3'

### *LmexCen*^*−/−*^ Immunization and challenge with needle infection

Six to eight-week-old female hamsters were immunized with 10^6^ total stationary-phase *LmexCen*^*−/−*^parasites by intradermal injection in the left ear in 10 μl PBS using a 29-gauge needle (BD Ultra-Fine). Eleven weeks post-immunization, animals were challenged with a needle injection (ID route) with virulent 5 × 10^5^ metacyclic *L. donovani* (Ld1S) promastigotes into the contralateral ear. Age-matched non-immunized hamsters were similarly challenged and used as a control group. Hamsters were monitored daily, and after various periods of post-challenge (9- and 15-month post-challenge), animals were sacrificed, and the parasite load in organs (spleen and liver) was measured by limiting dilution assay^27^.

### *LmexCen*^*−/−*^ Immunization and challenge with sand fly mediated infection

Female 2–4 days-old *Lutzomyia longipalpis* sand flies were infected with *L. donovani* amastigotes (freshly isolated from a terminally sick hamster due to visceral leishmaniasis). The sand flies were infected by artificial feeding through a chicken skin membrane on rabbit blood containing *Leishmania donovani* amastigotes. Fully blood fed female sand flies were separated and maintained at 26 °C with 75% humidity and were provided 30% sucrose solution. The flies were dissected periodically to assess the the maturity of infection^27^. Parasite loads and percentage of metacyclic per midgut were determined using hemocytometer counts. Thirty sand flies on day 15- after infection were applied to the ear of each *LmexCen*^***−/−***^ immunized and age-matched non-immunized hamsters used for subsequent transmission as described previously. The number of blood-fed flies was determined after transmission as a qualitative measurement. Each hamster received an average of 18 infected bites per transmission. Due to COVID related restrictions it was not possible to have separate control groups for *LmCen*^−/−^ and *LmexCen*^−/−^ studies that were conducted concurrently. Thus, the sand fly challenge experiments with non-immunized hamster controls shown in the current study were performed alongside our previous studies on *LmCen*^*−/−*^ parasites^27^. Hamsters were monitored daily during infection, and after 9–15-months of post-challenge, animals were sacrificed, and parasite load in organs (spleen and liver) was measured by limiting dilution assay^27^.

### Statistical analysis

Statistical analysis of differences between groups was determined by unpaired two-tailed Mann–Whitney *t*-test using Graph Pad Prism 7.0 software.

### Reporting summary

Further information on research design is available in the Nature Research Reporting Summary linked to this article.

## Supplementary information

REPORTING SUMMARY

## Data Availability

The data that support the findings of this study are available from the corresponding authors HLN or RD upon request.
